# Enhancing Mesopore Volume and Thermal Insulation of Silica Aerogel via Ambient Pressure Drying-Assisted Foaming Method

**DOI:** 10.3390/ma17112641

**Published:** 2024-05-30

**Authors:** Jinjing Guo, Kaiqiang Luo, Wenqi Zou, Jun Xu, Baohua Guo

**Affiliations:** Department of Chemical Engineering, Tsinghua University, Beijing 100084, China; gjj19@mails.tsinghua.edu.cn (J.G.); zouwenqi.2020@tsinghua.org.cn (W.Z.)

**Keywords:** silica aerogel, ambient pressure drying, spring-back, high mesopore volume, thermal conductivity

## Abstract

Ambient pressure drying (APD) of silica aerogels has emerged as an attractive method adapting to large-scale production. Spring-back is a unique phenomenon during APD of silica aerogels with volume expansion after its shrinkage under capillary force. We attribute the intense spring-back at elevated drying temperatures to a dense structure formed on the surface and the formation of positive internal pressure. Furthermore, an APD-assisted foaming method with an in situ introduction of NH_4_HCO_3_ was proposed. NH_4_HCO_3_ decomposing at drying temperatures hastened the emergence of positive pressure, thereby increasing the expansion volume. Compared to the previous method, the porosity of silica aerogel increased from 82.2% to 92.6%, and mesopore volume from 1.79 cm^3^ g^−1^ to 4.54 cm^3^ g^−1^. By adjusting the amount of the silicon source, silica aerogels prepared by the APD-assisted foaming method generated higher volume expansion and lower thermal conductivity. After calcination to remove undecomposed ammonium salts, the hydrophobic silica aerogel with a density of 0.112 g cm^−3^ reached a mesopore volume of 5.07 cm^3^ g^−1^ and a thermal conductivity of 18.9 mW m^−1^·K^−1^. This strategy not only improves the thermal insulation properties, but also offers a significant advancement in tailoring silica aerogels with specific porosity and mesopore volume for various applications.

## 1. Introduction

Silica aerogels are nanomaterials with a three-dimensional network structure and extremely high porosity, typically characterized by very high specific surface areas and extremely low densities [1,2]. Silica aerogels generally exhibit ultra-low thermal conductivities primarily dependent on pores in the order of tens of nanometers, which are smaller than the mean free path of air molecules, effectively blocking convective heat transfer through the Knudsen effect [3,4]. Additionally, the path for solid-phase heat transfer is provided by the stacked skeleton of silica secondary particles. The gel skeletons with diameters less than 10 nm significantly reduce the contribution of solid-phase heat transfer [5]. The formation of numerous interfaces also contributes to effective phonon scattering, further reducing thermal conductivity [6].

The preparation methods for silica aerogels generally include supercritical fluid drying, freeze-drying, and ambient pressure drying [1,7,8]. Supercritical fluid drying involves replacing the solvent in wet gels with supercritical fluid under certain temperatures and pressures. Then, an intact nano-sized gel skeleton can be obtained by reducing the pressure at a constant temperature [2]. However, supercritical fluid drying has limitations in cost efficiency, process continuity, and safety due to the high pressures required to reach the critical point [9]. Freeze-drying involves solidifying the solvent in the gel and then sublimating the solvent at relative low pressures [10,11]. The freeze-drying method is generally limited to laboratory-scale preparations. Ambient pressure drying, which involves replacing the internal solvent in hydrophobic-modified silica aerogels with a low-surface-tension solvent and drying under atmospheric conditions, offers a promising approach to reduce the production cost and improve production efficiency of silica aerogels [12,13,14].

The initial ambient pressure drying stage removes the bulk of the solvent from the silica gel, causing the volume to shrink as the capillary forces within the pores pull the skeleton inward. However, the gel expands extremely during subsequent drying, a phenomenon known as the spring-back effect, first observed by Prakash et. al. during the drying process of trimethylchlorosilane-modified silica aerogel [15]. Subsequent studies focusing on ambient pressure drying of hydrophobically modified silica aerogels have consistently observed significant spring-back. For instance, aerogels with methyltrimethoxysilane as the silicon source, which were initially dried slowly at room temperature for a day, leading to continuous shrinkage of the gel volume, exhibited the spring-back phenomenon after heating at 300 °C for 1 h [16,17]. Similarly, silica aerogels modified with methyltriethoxysilane and 3-methacryloxypropyltrimethoxysilane also displayed spring-back during a gradual temperature increase from 40 °C to 80 °C [18].

The reason for spring-back has been generally attributed to the recovery of elastic deformation upon the disappearance of capillary forces in pores of aerogels [13,15]. However, drying temperatures of ambient pressure drying have significant influence on the expansion volume of spring-back, which cannot be explained on the basis of elastic deformation. Silica aerogels dried slowly at room temperature typically exhibit significant volume shrinkage, resulting in densities exceeding 0.3 g cm^−3^ and thermal conductivities reaching above 30 mW m^−1^ K^−1^ [19,20,21,22], whereas for tetraethoxysilane- and methyltrimethoxysilane-based silica aerogels dried above the boiling point of the inner solvent, micropore volumes increased by 2.5 times and mesopore volumes by 3.0 times compared to room temperature drying [13]. Hydrophobic silica aerogels using sodium silicate precursors with various silylating agents, such as methyltrimethoxysilane, dimethyldimethoxysilane and trimethylmethoxy silane, also demonstrated a more than 60% volume rebound when dried above the solvent’s boiling point [21].

The volume expansion brought by spring-back is critical to overall thermal conductivity of silica aerogels, affected by both the porosity and the volume of mesopores. Lower porosity, implying a higher content of the gel skeleton per unit volume, enhances solid-phase heat transfer. Meanwhile, a certain volume of mesopores is crucial for blocking convective air heat transfer. The spring-back effect highlights the complex interplay between the drying processes of aerogels and their physical properties. Managing this effect is promising for optimizing the structure, microscopic morphology, and thermal insulation properties of silica aerogels. Inspired by the rapid formation of highly porous wing structures during dragonfly hatching in water, Han et al. [23] used a method where the hydrophobic modification solvent TMCS reacts with NaHCO_3_ at room temperature to in situ generate CO_2_ in nanopores. The resulting silica aerogel had a pore volume of 1.2 cm^3^ g^−1^ and an average pore diameter of 7 nm, with a thermal conductivity of 0.016 W m^−1^ K^−1^. However, this method requires ion exchange to remove the byproduct NaCl. Subsequently, Lu et al. [24] explored the introduction of nickel nanoparticles into silica aerogels prepared by ambient pressure drying, varying the proportion of in situ introduced NH_4_HCO_3_ to control the BET specific surface area and porosity. Although the pore volume varied within the small range of 0.72–0.87 cm^3^ g^−1^, they offered new perspectives on enhancing the porosity of silica aerogels with easy removal of byproducts.

Herein, an APD-assisted foaming method was introduced to show the capability of effectively increasing the expansion volume during spring-back. This method not only barely affects the size of silica secondary particles constituting the gel skeleton, but also increases the mesopore volume and overall porosity of silicon aerogels. The APD-assisted foaming method significantly decreases thermal conductivities of silica aerogels compared to traditional methods. According to the results of this strategy, we proposed a novel explanation for the spring-back phenomenon observed in hydrophobic silica aerogels dried above the solvent’s boiling point under ambient pressure.

## 2. Materials and Methods

### 2.1. Materials

Tetraethoxysilane (TEOS, 99%), chlorotrimethylsilane (TMCS, 98%), and ammonium bicarbonate (NH_4_HCO_3_, 99%) were purchased from Innochem Science & Technology Co., Ltd., Beijing, China. Ethanol (EtOH, 99.5%), N, N-dimethylformamide (DMF, 99.5%), isopropanol (i-PrOH, 99.7%), and n-hexane (n-Hex, 97.0%) were purchased from Shanghai Aladdin Biochemical Technology Co., Ltd., Shanghai, China. Hydrochloric acid (HCl, AR) and ammonia water (with 25 wt% ammonia dissolved) were purchased from Beijing Chemical Factory, Beijing, China. All the chemicals were used without further purification.

### 2.2. Preparation of Granular Silica Aerogels (1 eqv, 0.75 eqv, and 0.5 eqv) with APD-Assisted Foaming Method

**Figure 1:**
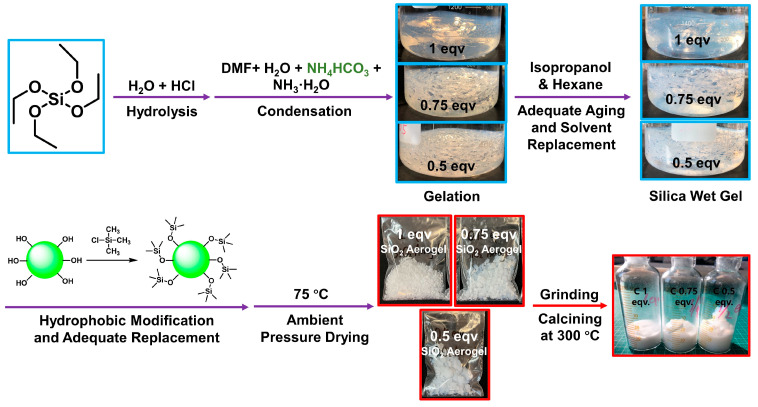
A process diagram for the preparation of silica aerogel based on the APD-assisted foaming method. The samples are named as 1 eqv, 0.75 eqv, and 0.5 eqv depending on the content of tetraethoxysilane (TEOS) as the silicon source. After sufficient hydrolysis–condensation of TEOS, solvent replacement with a gradient of isopropanol to n-hexane was followed. Subsequently, the gels underwent hydrophobic modification with trimethylchlorosilane and solvent replacement with n-hexane. Finally, the granular silica aerogels were obtained with sufficient drying at 75 °C. After grinding and calcining in a muffle furnace at 300 °C to completely remove undecomposed ammonium salts, corresponding silica aerogels are named as C-1 eqv, C-0.75 eqv, and C-0.5 eqv, respectively.

The procedure is outlined in Figure 1. In three 2000 mL glass beakers, the following mixtures were prepared: 141.8 mL of TEOS with 258.4 mL of EtOH, 115.1 mL of TEOS with 279.6 mL of EtOH, and 83.5 mL of TEOS with 304.5 mL of EtOH. These samples were designated as 1 eqv, 0.75 eqv, and 0.5 eqv, respectively. These mixtures were stirred at room temperature for 30 min. To 200 mL of deionized water, HCl was added to adjust the pH of the acidic aqueous solution to 3.85. Using a 5000 μL pipette, 45.58 mL, 49.31 mL, and 53.70 mL of this acidic aqueous solution were added to the 1 eqv, 0.75 eqv, and 0.5 eqv samples, respectively. After being sealed with three layers of low-density polyethylene thin film, three beakers were placed in a 35 °C water bath. It was essential to ensure that the water bath completely submerged the content of each beaker. These mixtures were stirred at 35 °C for 48 h.

After 48 h, 12.25 mL, 13.26 mL, and 14.44 mL of DMF were slowly added to the 1 eqv, 0.75 eqv, and 0.5 eqv samples, respectively, followed by vigorous stirring for 60 min. A layer of polyimide film covered the inner wall of each beaker to facilitate the demolding of the gel. Ammonia water was added to 100 mL of deionized water to prepare an alkaline aqueous solution with a pH of 11.12. To this alkaline aqueous solution, 7.50 g of NH_4_HCO_3_ was added. Stirring slowly at room temperature was conducted until NH_4_HCO_3_ was completely dissolved without bubbles. Then, 22.79 mL, 24.65 mL, and 26.85 mL of the prepared NH_4_HCO_3_ alkaline solution were added to the 1 eqv, 0.75 eqv, and 0.5 eqv samples under stirring conditions. After stirring for 2 min, magnetic stirrers were removed, and the solutions were left to gelation. Gelation occurred within 5–20 min for all three samples. The beakers were then sealed with three layers of low-density polyethylene thin film and left to age in a 35 °C water bath for 24 h. During this period, the gel samples underwent volume shrinkage. After removing the liquid from the beakers, the polyimide films were removed to demold wet gels. During the preparation process, the molar ratios of raw materials for the preparation of silica aerogels were TEOS:H_2_O:EtOH:DMF:HCl:NH_3_·H_2_O = 1/0.75/0.5:6:7:0.25:10^−5^:3.57 × 10^−3^.

Each beaker was then filled with 1200 mL of a deionized water/EtOH mixture at a volume ratio of 1:4, containing 18 g of NH_4_HCO_3_. After sealing the beakers, wet gels were placed in a 35 °C water bath for 48 h of aging. Then, the aging liquid was discarded, and each sample underwent solvent exchange for 5 rounds. Each solvent exchange underwent a 35 °C water bath for 24 h, with 800 mL of an i-PrOH and n-Hex mixture at volume ratios of 100/0, 75/25, 50/50, 25/75, and 0/100, respectively. Finally, the n-Hex was poured out to complete the solvent exchange for the gels.

In total, 240 mL of TMCS was slowly added to 2160 mL of n-Hex with thorough stirring. This solution was divided into three equal volumes and added to the 1 eqv, 0.75 eqv, and 0.5 eqv samples, respectively. The samples were left in a ventilated environment for 24 h for hydrophobic modification. After pouring out the solution, a new batch of the aforementioned hydrophobic modification solution was prepared and added to the samples for the other 24 h. All solutions were then removed, and 800 mL of n-Hex was added to each sample for solvent exchange to remove excess TMCS. For each solvent exchange, the beakers were sealed and left for 24 h. The solvent exchange step should be repeated 3 times. The gel samples were then placed in a 75 °C explosion-proof oven for thorough ambient pressure drying for 48 h, resulting in silica aerogels named 1 eqv, 0.75 eqv, and 0.5 eqv, respectively.

### 2.3. Preparation of Calcined Silica Aerogels (C-1 eqv, C-0.75 eqv, and C-0.5 eqv) to Remove Residual Ammonium Salts

Based on the results from the thermogravimetric analyzer, the initial decomposition temperature of NH_4_HCO_3_ is approximately 70 °C, and that of NH_4_Cl is around 170 °C, as shown in Figure S1. Consequently, the silica aerogel samples, 1 eqv, 0.75 eqv, and 0.5 eqv, were ground into powder using a mortar and placed in a muffle furnace. The temperature was set to 300 °C. The samples were calcined for 3 h. Afterwards, heating was turned off and cooling to room temperature took place. Corresponding calcined silica aerogels were named as C-1 eqv, C-0.75 eqv, and C-0.5 eqv, respectively.

### 2.4. Preparation of General Silica Aerogel (Un-1 eqv) via Ambient Pressure Drying as the Comparison Group

For comparison, the operations of 1 eqv, 0.75 eqv, and 0.5 eqv TEOS hydrolyzing under acidic conditions at 35 °C for 48 h were identical to 2.2.

After 48 h, 12.25 mL, 13.26 mL, and 14.44 mL of DMF were slowly added to the 1 eqv, 0.75 eqv, and 0.5 eqv samples, respectively, followed by vigorous stirring for 60 min. A layer of polyimide film covered the inner wall of each beaker to facilitate the demolding of gels. Ammonia water was added to 100 mL of deionized water to prepare an alkaline aqueous solution with a pH of 11.12. However, only the 1 eqv sample completed gelation within 1 h; the other two samples could not gel within 3 h. The 0.75 eqv sample, which gelled within 8 h, was too weak to separate the gel from the solution during solvent exchange. So, only the 1 eqv sample was kept for subsequent operations, which was renamed as the Un-1 eqv sample.

The beaker was then sealed with three layers of low-density polyethylene thin film and left in a 35 °C water bath for 24 h. After pouring out the liquid from the beaker and removing the polyimide film to demold, 1200 mL of a 1:4 volume ratio of a deionized water/EtOH solution was added. The beakers were sealed again and aged in a 35 °C water bath for 48 h. After aging, liquid was removed, and the Un-1 eqv sample underwent 5 rounds of solvent exchange in a 35 °C water bath with 800 mL each of i-PrOH and n-Hex mixtures at volume ratios of 100/0, 75/25, 50/50, 25/75, and 0/100, respectively. Solvent exchange completed 24 h before proceeding to the next round. Finally, the n-Hex was poured out to complete the solvent exchange for the Un-1 eqv sample.

To 720 mL of n-Hex, 80 mL of TMCS was slowly added and thoroughly stirred. This solution was then added to the Un-1 eqv sample. The sample was left in a ventilated environment for 24 h for hydrophobic modification. After pouring out the solution, a new batch of the TMCS/n-Hex solution was prepared and added to Un-1 eqv for the other 24 h of thorough hydrophobic modification. The solution was then discarded, and 800 mL of n-Hex was added for solvent exchange to remove excess TMCS. For each solvent exchange, the beaker was sealed and left for 24 h. The solvent exchange step should be repeated 3 times. The Un-1 eqv gel sample was then removed and placed in a 75 °C explosion-proof oven for thorough ambient pressure drying for 48 h, resulting in the final silica aerogels named Un-1 eqv.

### 2.5. Characterization and Measurement

The pH values of water solutions were measured by a Sartorius PB-10 standard pH meter adopting the three-point calibration. Fourier transform infrared (FT−IR) spectra were collected employing a Thermo Scientific Nicolet 6700 spectrometer (Waltham, MA, USA). The measurements involved signal averaging over 32 scans at a resolution of 4 cm^−1^ in the wavenumber range of 650–4000 cm^−1^. Prior to the analysis, samples were ground to fine powder using an agate mortar and then mixed with KBr. Scanning electron microscopy (SEM) imaging was conducted using a JEOL JSM 7800f instrument (Akishima shi, Japan) operating at an accelerating voltage of 5.0 kV. Transmission electron microscopy (TEM) imaging was acquired with a JEOL 1011 operating at 200 kV. Adsorption–desorption isotherms of Nitrogen (N_2_) were recorded at −196 °C on a PULSAR TPR/T Quantachrome Chembet (Boynton Beach, FL, USA). All samples underwent vacuum degassing for 48 h at 40 °C. Specific surface areas were determined using the Brunauer, Emmett, and Teller (BET) method in the relative pressure range of 0.05–0.3. Pore size distributions and pore volumes were computed using N_2_ adsorption at 77 K, following the N_2_ at 77 K in the silica (cylinder pore, nonlocal density functional theory adsorption branch) model. Water contact angles were measured at room temperature (25 °C) using a HARKE HKCA-10 contact angle tester (Beijing, China). The appearance photos and the time-lapse video of ambient pressure drying were recorded by an iPhone SE. Thermal conductivity was assessed at room temperature (25 °C), employing a Hot Disk TPS 2500s thermal constants analyzer (Gothenburg, Sweden). Apparent densities were determined following ISO 1183-1-2019 standards [25]. Each measurement was performed at least 3 times to ensure accuracy. Thermalgravimetric curves were detected by a Shimadzu DTG-60 thermogravimetric analyzer (Kyoto, Japan) under 50 mL min^−1^ N_2_ protection, of which temperature ranges were set from room temperature to 700 °C and heating rates were set to 20 °C min^−1^.

## 3. Results and Discussion

### 3.1. Chemical Structures of Silica Aerogels Prepared by the APD-Assisted Foaming Method

**Figure 2:**
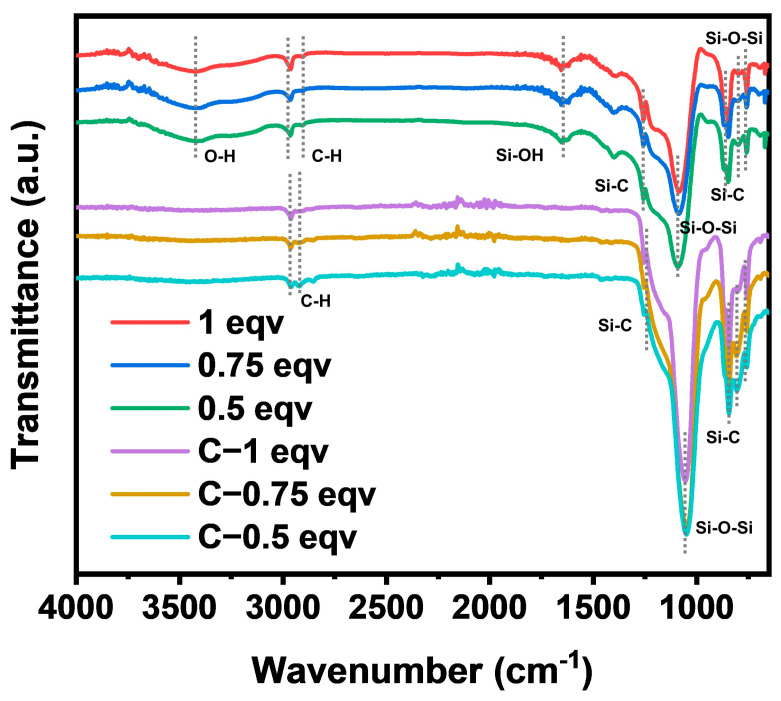
FT−IR spectra of silica aerogels.

The chemical structure of each silica aerogel was analyzed by FT−IR spectra, as shown in Figure 2. The positions and intensities of the absorption peaks of 1 eqv, 0.75 eqv, and 0.5 eqv were essentially the same, indicating that silica aerogels had similar chemical structures. Strong absorption peaks for Si-O-Si were located at 1088 cm^−1^ and 780 cm^−1^ [26]. Stretching vibrations of Si-C bonds were found at 1255 cm^−1^, 847 cm^−1^, and 759 cm^−1^. Symmetric and asymmetric stretching vibrations of C-H bonds at 2961 cm^−1^ and 2904 cm^−1^ indicated the presence of Si-CH_3_ [27,28], suggesting that the gel underwent hydrophobic modification. However, a broad peak at 3440 cm^−1^ for OH stretching vibrations [29] and a peak at 1659 cm^−1^ for Si-OH bending vibrations indicated the presence of Si-OH or adsorbed water within the silica aerogels [30], suggesting that the hydrophobic modification might not be sufficient. After calcination at 300 °C, C-1 eqv, C-0.75 eqv, and C-0.5 eqv aerogels showed significant reduction in Si-OH and OH absorption peaks, indicating removal of adsorbed water and the potential conversion of Si-OH bonds to Si-O-Si bonds. The corresponding increase in the absorption peaks for Si-O-Si bonds and the more pronounced stretching vibrations for Si-C bonds suggested that the main component of the gel skeleton did not undergo significant changes during calcination.

### 3.2. Morphology of the Skeleton and Pores of Silica Aerogels Prepared by the APD-Assisted Foaming Method

**Figure 3:**
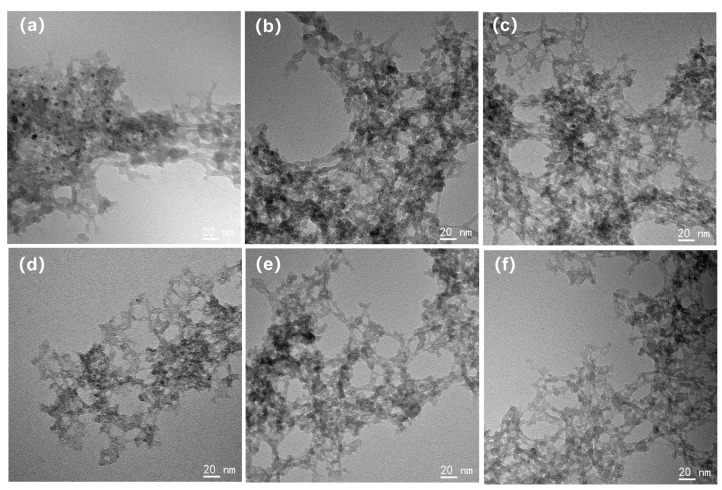
TEM images of silica aerogels; (**a**) 1 eqv, (**b**) 0.75 eqv, (**c**) 0.5 eqv, (**d**) C-1 eqv, (**e**) C-0.75 eqv, (**f**) C-0.5 eqv. The sizes of silica secondary particles are all less than 10 nm and relatively uniform.

The TEM morphology of each silica aerogel is depicted in Figure 3. It should be noted that the TEM images display the morphology of the silica aerogels redispersed on ultrathin carbon films. The sample preparation process would not affect the size of silica secondary particles, but due to the capillary tension, the size of the pores within gels had changed. For the 1 eqv, 0.75 eqv, and 0.5 eqv silica aerogels prepared by the APD-assisted foaming method, similar to the Un-1 eqv skeleton, the size of the secondary particles composing the gel skeleton was 10 nm or below. This uniformity in particle size was mainly because TEOS was used as the only silicon source and underwent similar solvent environments and temperatures during the hydrolysis–condensation reactions, resulting in relatively small variations in the size of the secondary silica particles [31]. After calcination to remove residual ammonium salts, the morphology of the C-1 eqv, C-0.75 eqv, and C-0.5 eqv silica aerogels did not show significant changes.

**Figure 4:**
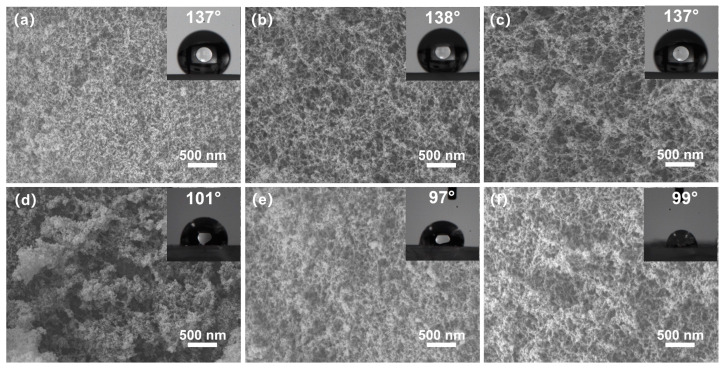
SEM images and contact angle images of silica aerogels; (**a**) 1 eqv, (**b**) 0.75 eqv, (**c**) 0.5 eqv, (**d**) C-1 eqv, (**e**) C-0.75 eqv, (**f**) C-0.5 eqv. No significant change in the morphology of gel skeletons before and after calcination was observed. All silica aerogels obtained high porosity and nanoscale pores.

The SEM morphology of the cross-sections of the silica aerogels is shown in Figure 4. The 1 eqv, 0.75 eqv, and 0.5 eqv silica aerogels had pores of several tens of nanometers. Finer skeleton morphology aligned with the results from the TEM images. The 0.75 eqv silica aerogel showed an increased porosity compared with 1 eqv. Similarly, the 0.5 eqv silica aerogel showed an increased porosity compared with 0.75 eqv. The calcined silica aerogels, C-1 eqv, C-0.75 eqv, and C-0.5 eqv, still possess nanoscale pores and high porosities. After calcination, the densities of C-1 eqv, C-0.75 eqv, and C-0.5 eqv become 96.6%, 95.6%, and 95.8% of the original one according to thermogravimetric curves shown in Figure S2, indicating that the original silica aerogels contained similar amounts of undecomposed ammonium salts.

Additionally, the water contact angles of 1 eqv, 0.75 eqv, and 0.5 eqv silica aerogels were approximately 137°. After calcination, the water contact angles of C-1 eqv, C-0.75 eqv, and C-0.5 eqv were around 100°. The change in the water contact angle could be attributed to the decomposition of Si-CH_3_ groups on the silica aerogel surface at 300 °C, resulting in the formation of the silicon surface or Si-O-Si bonds.

### 3.3. Analysis of Mesopores and Thermal Conductivities of Silica Aerogels Prepared by the APD-Assisted Foaming Method

**Figure 5:**
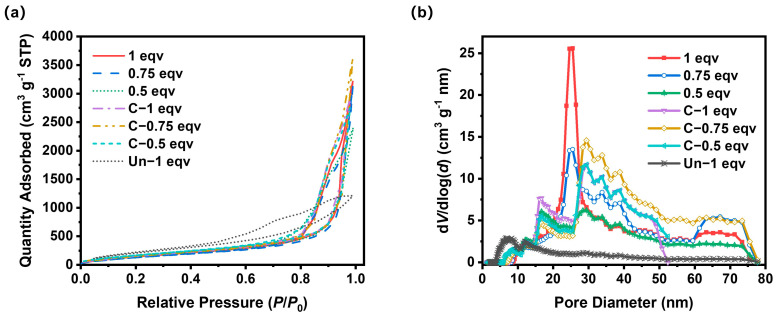
(**a**) N_2_ adsorption−desorption isotherm curves for silica aerogels. (**b**) Pore size distribution curves of silica aerogels.

To further analyze the effect of the APD-assisted foaming method on the pore structures of silica aerogels, especially on mesopores, N_2_ adsorption–desorption tests were performed, as shown in Figure 5a. According to IUPAC classifications, the N_2_ adsorption–desorption isotherms of all silica aerogels belong to Type V isotherms with H3 hysteresis loops. The absence of adsorption saturation and the high adsorption volumes indicate that the silica aerogels produced by the APD-assisted foaming method possess slit pore structures. A small area of hysteresis loops corresponded with a good openness of inner pores. The BET specific surface areas of all samples were within the range of 580–700 m^2^ g^−1^. After calcination, the BET specific surface areas slightly increased, suggesting that undecomposed ammonium salts also existed in mesopores within 100 nm. The pore size distribution curves for all samples, as depicted in Figure 5b, show that 1 eqv silica aerogel had a higher mesopore volume compared to 0.75 eqv and 0.5 eqv, with a peak of pores around 24 nm in diameter. The mesopore volume of 0.75 eqv was 93.8% of 1 eqv, but its apparent density was only 85.9% of 1 eqv, indicating that the reduction in the content of the silicon source and drying process led to the formation of macropores over 100 nm during the spring-back. This was further confirmed by the results for 0.5 eqv, whose mesopore volume was 79.8% of 0.75 eqv, while its apparent density only dropped to 85.2% of 0.75 eqv.

After calcination, the mesopore volume of C-1 eqv decreased to 4.17 cm^3^ g^−1^ compared with 4.54 cm^3^ g^−1^ of 1 eqv, primarily indicated by the disappearance of the peak at 24 nm in diameter. After the complete decomposition of ammonium salts, the mesopore volume for diameters between 30 and 50 nm increased. But it likely also resulted in the formation of macropores over 100 nm, which corresponds to a decrease in mesopore volume with a limited increase in BET specific surface area shown in Table 1. However, for 0.75 eqv and 0.5 eqv, which already had more macropores over 100 nm during the spring-back process, the calcined C-0.75 eqv and C-0.5 eqv showed a mesopore volume increase of 19.0% and 16.2%, respectively, while the decrease in apparent density was 10.2% and 14.8%, respectively, according to data in Table 1. C-0.75 eqv showed an increase in the content of mesopores between 30 and 80 nm in diameter, and C-0.5 eqv showed an increase in the distribution of mesopores between 30 and 50 nm in diameter.

In stark contrast to the silica aerogels prepared by the APD-assisted foaming method, the Un-1 eqv had relatively low saturation adsorption volume. Although it possessed a high BET specific surface area up to 879 m^2^ g^−1^, which was mostly contributed by mesopores below 25 nm, the mesopore volume was limited to 1.79 cm^3^ g^−1^ due to smaller volume expansion. The mesopore volume of C-1 eqv was 2.32 times that of Un-1 eqv. The density of Un-1 eqv was 2.57 times that of C-1 eqv, indicating that the APD-assisted foaming method effectively enhanced the overall porosity of silica aerogels, especially significantly increasing the mesopore volume.

**Figure 6:**
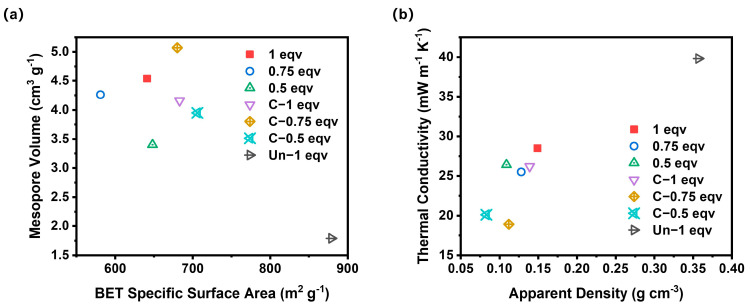
(**a**) A comparation plot of BET specific surface area and mesopore volume of silica aerogels. (**b**) A comparation plot of apparent density and thermal conductivity of silica aerogels.

To analyze the impact of various factors on the thermal conductivities of silica aerogels, BET specific surface area, mesopore volume, apparent density, and thermal conductivity of all aerogel samples were compared simultaneously, as shown in Figure 6. Among them, C-0.75 eqv exhibited the lowest thermal conductivity of 18.9 mW m^−1^ K^−1^. This is mainly due to the volume of mesopores distributed below 69 nm obstructing convective heat transfer, and the lower density reducing the solid-phase heat transfer within the aerogel. If the silica aerogels are ranked solely based on their mesopore volume, the thermal conductivity from lowest to highest would be C-0.75 eqv, 1 eqv, 0.75 eqv, C-1 eqv, C-0.5 eqv, and 0.5 eqv. However, the actual thermal conductivity from lowest to highest is C-0.75 eqv, C-0.5 eqv, 0.75 eqv, C-1 eqv, 0.5 eqv, and 1 eqv. The most significant deviations from the mesopore volume ranking are observed with C-0.5 eqv and 1 eqv, which is attributed to differences in the stacking degree of the gel skeleton. Higher porosity with a more dispersed gel skeleton contributes less to solid-phase heat transfer. Correspondingly, C-0.5 eqv silica aerogel had the lowest density among all samples, at 0.083 g cm^−3^, whereas 1 eqv, with a higher silicon source content and undecomposed ammonium salts within its pores, had a density as high as 0.149 g cm^−3^. Even though 1 eqv had a relatively higher mesopore volume, its thermal conductivity only reached 28.5 mW m^−1^ K^−1^. In contrast, Un-1 eqv silica aerogel had a thermal conductivity as high as 39.8 mW m^−1^ K^−1^, further illustrating the effectiveness of the APD-assisted foaming method in enhancing the expansion volume of spring-back, thereby reducing both solid-phase heat transfer and air convection within the aerogel, achieving remarkable thermal insulation properties.

### 3.4. Explanations for Spring-Back Phenomenon and the APD-Assisted Foaming Method

Finally, we propose a possible theoretical explanation for the spring-back phenomenon observed during the ambient pressure drying process of silica aerogels at elevated temperatures above the boiling point of the inner solvent.

**Figure 7:**
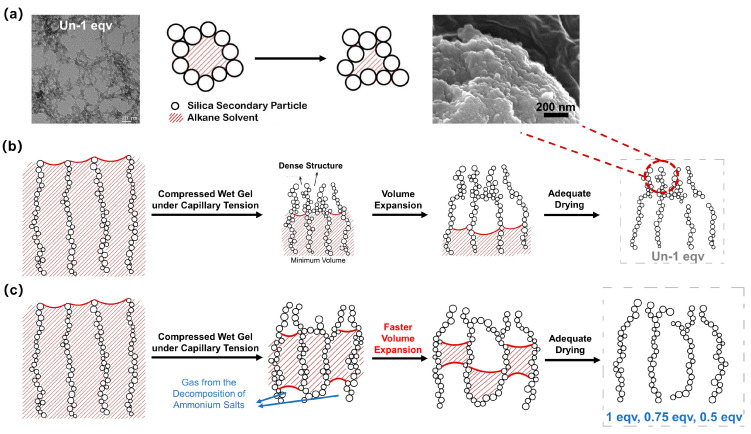
Schematic diagrams of the changes in pore structure of silica aerogels during ambient pressure drying at elevated temperatures above the boiling point of the inner solvent. (**a**) A schematic diagram of the variable stacking of silica secondary particles during the ambient drying process. A TEM image of Un-1 eqv shows the point-to-point connecting of secondary particles. (**b**) A schematic diagram of changes in pore structure of general silica aerogel during ambient pressure drying at elevated temperatures. (**c**) A schematic diagram of changes in pore structure of silica aerogels with high porosity prepared by the APD-assisted foaming method.

As illustrated in Figure 7a, TEM images indicate that the skeleton of silica aerogel consists of point-to-point contacts between silica secondary particles, or the secondary particles form a pearl necklace-like structure stacked together during the aging process. Notably, the gel skeleton is not rigid, due to the presence of the solvent and good wettability conditions [9]. The stacking variability of silica secondary particles macroscopically manifests as volume shrinkage and expansion during the ambient pressure drying process.

As shown in Video S1, for the Un-1 eqv silica wet gel, the volume slightly expanded after shrinking to its minimum during the drying process. The gel skeleton initially shrank under the compression of capillary tension. Then, a dense structure was gradually formed on the surface. The surface SEM morphology of the Un-1 eqv silica aerogel, as shown in Figure 7b, reveals a dense structure. Since the surface layer of wet gels was affected by capillary tension of the internal solvent for an extended period during the ambient pressure drying, the variable stacking of secondary particles under compression stress leads to the formation of a dense structure. The vaporized solvent could not discharge promptly from the gel skeleton during the subsequent drying process. Thus, as the internal solvent of the gel continues to evaporate without exiting the pores promptly, a positive pressure environment is formed within the gel pores. The volume expansion of the gel happens, known as the spring-back effect. Granular silica aerogels can be obtained after a thorough evaporation of the solvent. It is worth noting that the spring-back effect can significantly affect the thermal conductivity. Less volume expansion implies lower porosity and a closer accumulation of silica secondary particles, thereby diminishing the effectiveness of blocking air convection heat transfer and solid heat transfer.

Based on the analysis of the reasons behind the spring-back effect, an APD-assisted foaming method for enhancing the spring-back effect is depicted in Figure 7c. By introducing NH_4_HCO_3_ into the silica sol, NH_4_HCO_3_ can be evenly dispersed within the gel pores upon gelation. The drying temperature for ambient pressure drying is set at 75 °C, at which the NH_4_HCO_3_ within the pores starts to decompose and generate gas. This process creates a positive pressure environment conducive to spring-back. As shown in Video S1, the 1 eqv silica aerogel made by the APD-assisted foaming method exhibited spring-back earlier and with a greater volume expansion compared with Un-1 eqv. Compared to the original preparation method without NH_4_HCO_3_, 0.75 and 0.5 equivalents of TEOS completed gelation within a short period, and both gels had certain strength to withstand subsequent procedures. This is mainly oriented from increasing the electrolyte concentration in a colloidal dispersion that compresses the electrical double layer around the particles [33]. The silica secondary particles are more prone to coagulate due to the attractive force between uncharged particles and the reduced repulsive barrier.

## 4. Conclusions

In conclusion, we have proposed a simple and effective APD-assisted foaming method to significantly increase the volume expansion of the spring-back effect during the preparation of silica aerogels. By introducing NH_4_HCO_3_ into the gel pores before gelation, the gels with TEOS contents of 0.75 eqv and 0.5 eqv obtained certain strength to withstand solvent exchange. After sufficient hydrophobic modification and solvent exchange, the decomposition of ammonium salts within the gel pores at ambient drying pressure accelerated the formation of a positive pressure environment inside gel pores, significantly increasing both porosity and mesopore volume of the silica aerogels. After calcination to remove the incompletely decomposed ammonium salts, the resulting aerogels had obtuse water contact angles. The size of silica secondary particles was 10 nm or below. The lowest thermal conductivity of the silica aerogels reached 18.9 mW m^−1^ K^−1^, with a density of 0.112 g cm^−3^ and a mesopore volume of 5.07 cm^3^ g^−1^. This simple method has broad application prospects in improving the porosity and mesopore volume of silica aerogels prepared by ambient pressure drying with hydrophobic silicon sources such as trimethoxymethylsilane, hexamethyldisiloxane, and so on. Given the potential explanation for spring-back commonly observed in ambient pressure drying, designing new types of silanes with small secondary particle sizes, high mechanical strength, and the ability to inhibit dramatic volume changes leading to fracturing in aerogels could pave the way for the ambient pressure drying of high-strength silica aerogel blocks.

## Figures and Tables

**Table 1:** Physical properties of silica aerogels.

Sample Name	*ρ* ^a^ (g cm^−3^)	Porosity ^b^ (%)	BET Specific Surface Area (m^2^ g^−1^)	Mesopore Volume ^c^ (cm^3^ g^−1^)	Average Pore Diameter ^d^ (nm)	Th.Conductivity ^e^ (mW m^−1^ K^−1^)
1 eqv	0.149 ± 0.007	92.6	641	4.54	25.5	28.5 ± 0.3
0.75 eqv	0.128 ± 0.013	93.6	581	4.26	25.5	25.5 ± 0.2
0.5 eqv	0.109 ± 0.009	94.6	648	3.40	29.4	26.4 ± 0.2
C-1 eqv	0.139 ± 0.006	93.1	683	4.16	29.4	26.2 ± 0.3
C-0.75 eqv	0.112 ± 0.009	94.4	680	5.07	29.4	18.9 ± 0.2
C-0.5 eqv	0.083 ± 0.014	95.9	706	3.95	29.4	20.1 ± 0.2
Un-1 eqv	0.357 ± 0.006	82.2	879	1.79	6.8	39.8 ± 0.1

^a^ Apparent density. ^b^ Porosity calculated by 1 − (*ρ*/*ρ*_skeleton_). *ρ*_skeleton_ was approximated by 2.0 g cm^−3^ [32]. ^c^ Mesopore volume calculated from N_2_ absorption measurements by NLDFT method. ^d^ Average pore diameter enumerated from N_2_ absorption measurements by NLDFT method. ^e^ Thermal conductivity measured by Hot Disk TPS 2500s thermal constants analyzer.

## Data Availability

Data are contained within the article and Supplementary Materials.

## Supplementary Materials

The following supporting information can be downloaded at: https://www.mdpi.com/article/10.3390/ma17112641/s1, Figure S1: Thermogravimetric curves for ammonium chloride and ammonium bicarbonate at a heating rate of 20 °C min^−1^; Figure S2: Thermogravimetric curves for silica aerogels at a heating rate of 20 °C min^−1^; Figure S3: (a) N_2_ adsorption−desorption isotherm curves for silica aerogels with various molar ratio of NH_4_HCO_3_ and without DMF. (b) Pore size distribution curves of silica aerogels with various molar ratio of NH_4_HCO_3_ and without DMF; Figure S4: A SEM image of UnDMF−1 eqv silica aerogel; Figure S5: A cross-sectional SEM image of Un-1 eqv silica aerogel; Table S1: Physical properties of silica aerogels with various molar ratio of NH_4_HCO_3_ and without DMF; Video S1: Time-lapse movie of spring-back during ambient pressure drying of Un-1 eqv and 1 eqv at 75 °C.
