# Supplementary material for: Enhancing Mesopore Volume and Thermal Insulation of Silica Aerogel via Ambient Pressure Drying-Assisted Foaming Method
Source: Materials (Basel). 2024 May 30;17(11):2641. doi: 10.3390/ma17112641 (PMC11173452; doi:10.3390/ma17112641)
Supplement: Supplementary file 1 [file materials-17-02641-s001.zip › Supporting Information for Publication.pdf]

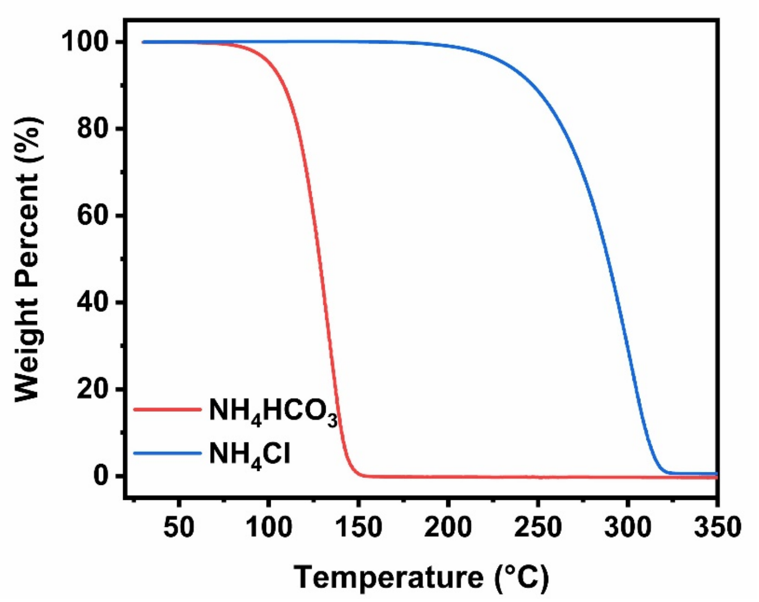

Figure S1. Thermogravimetric curves for ammonium chloride and ammonium bicarbonate at a heating rate of 20 °C min^{-1}.

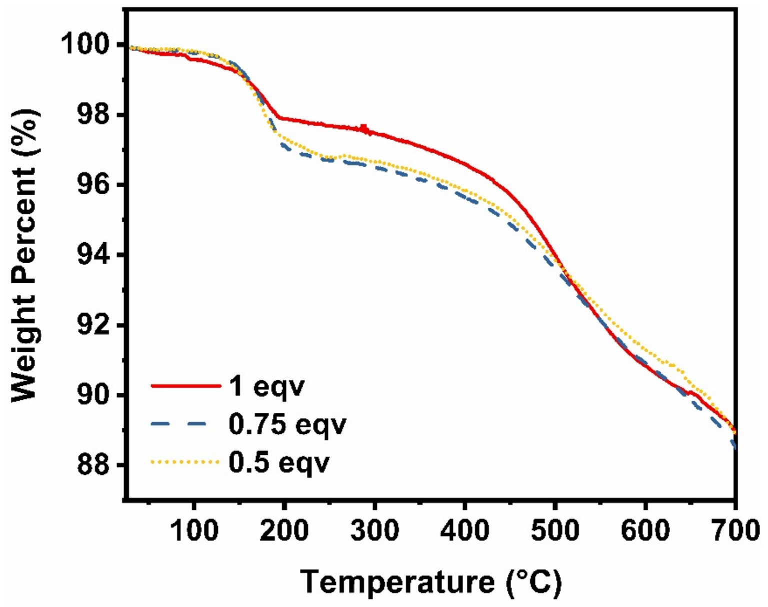

Figure S2. Thermogravimetric curves for silica aerogels at a heating rate of 20 °C min^{-1}.

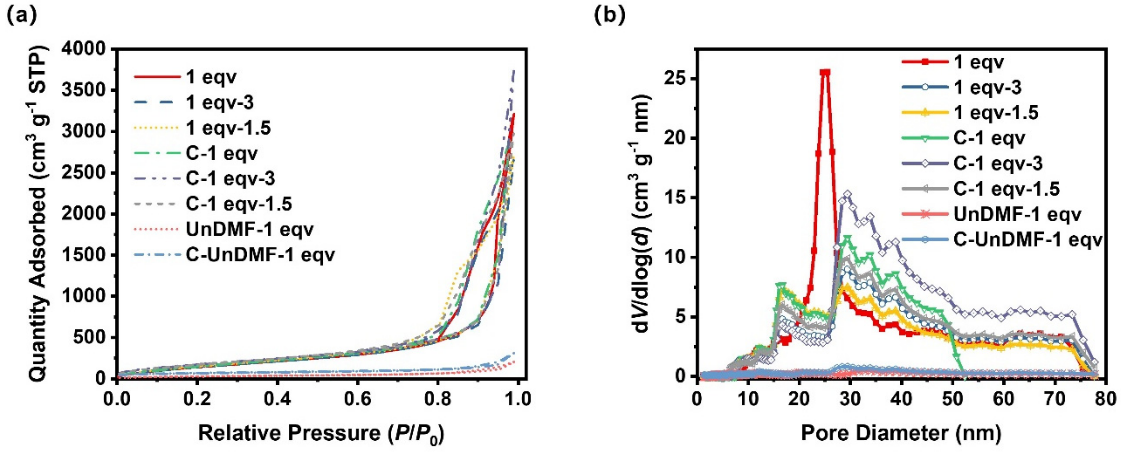

Figure S3. (a) N_2 adsorption−desorption isotherm curves for silica aerogels with various molar ratio of NH_4HCO_3 and without DMF. (b) Pore size distribution curves of silica aerogels with various molar ratio of NH_4HCO_3 and without DMF.

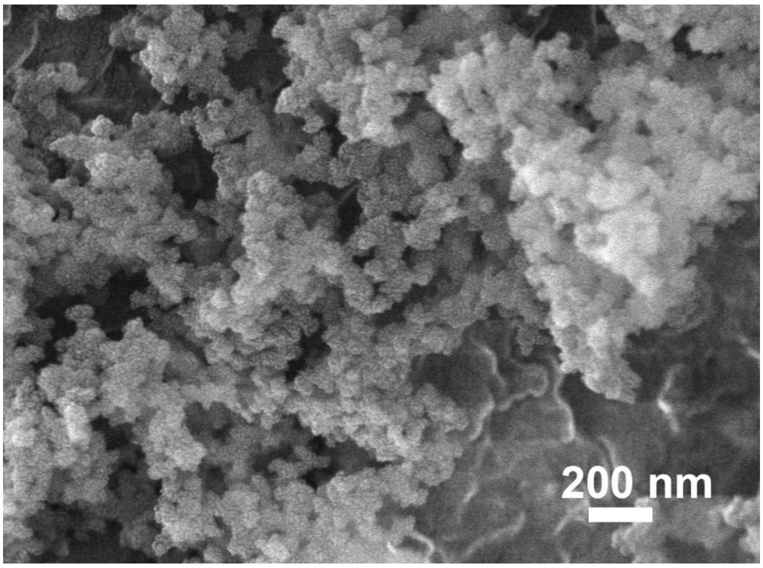

Figure S4. A SEM image of UnDMF−1 eqv silica aerogel.

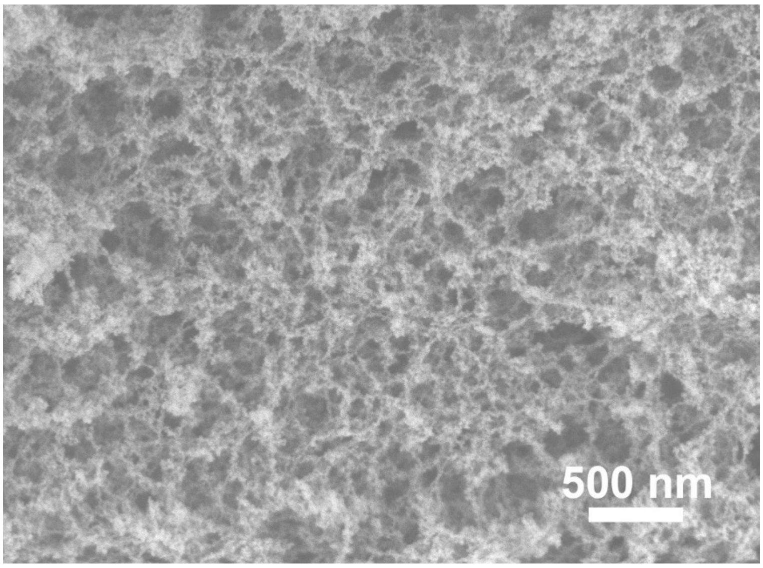

Figure S5. A cross-sectional SEM image of Un−1 eqv silica aerogel.

Table S1. Physical properties of silica aerogels with various molar ratio of NH_4HCO_3 and without DMF..

Sample Name	ρ^a (g cm^{-3})	Porosity^b (%)	BET Specific Surface Area (m^2 g^{-1})	Mesopore Volume^c (cm^3 g^{-1})	Average Pore Diameter^d (nm)	Th.Conductivity^e (mW m^{-1} K^{-1})
1 eqv	0.149 ± 0.007	92.6	641	4.54	25.5	28.5 ± 0.3
1 eqv−3	0.153 ± 0.006	92.4	626	3.76	29.4	28.2 ± 0.6
1 eqv−1.5	0.152 ± 0.006	92.4	659	3.89	29.4	28.3 ± 0.4
C−1 eqv	0.139 ± 0.006	93.1	683	4.16	29.4	26.2 ± 0.3
C−1 eqv−3	0.139 ± 0.012	93.1	693	4.19	29.4	25.8 ± 0.2
C−1 eqv−1.5	0.141 ± 0.008	93	702	4.25	29.4	26.1 ± 0.3
UnDMF−1 eqv	/	/	104	0.29	33.8	/
C−UnDMF−1 eqv	/	/	230	0.45	29.4	/

^{a}Apparent density. ^{b}Porosity calculated by $1-(\rho/\rho_{skeleton})$. $\rho_{skeleton}$ was approximated by 2.0 g cm^{-3}. ^{c}Mesopore volume calculated from N_2 absorption measurements by NLDFT method. ^{d}Average pore diameter enumerated from N_2 absorption measurements by NLDFT method. ^{e}Thermal conductivity measured by a Hot Disk TPS 2500s thermal constants analyzer.
